# Potential of Current Direct Mechanical Testing Methods in Assessing Intraoperative Samples of Aortic Aneurysm Caused by Uncontrolled Arterial Hypertension

**DOI:** 10.17691/stm2024.16.4.05

**Published:** 2024-08-30

**Authors:** V.N. Nikolenko, Y.V. Belov, M.V. Oganesyan, Y.M. Efremov, N.A. Rizaeva, A.D. Vovkogon, A.V. Sankov, L.A. Gridin, P.S. Timashev, K.V. Bulygin, M.V. Sankova

**Affiliations:** MD, PhD, Professor, Head of the Human Anatomy and Histology Department; I.M. Sechenov First Moscow State Medical University (Sechenov University), 8/2 Trubetskaya St., Moscow, 119991, Russia; Head of the Normal and Topographic Anatomy Department, Fundamental Medicine Faculty; Lomonosov Moscow State University, 1 Leninskiye Gory, Moscow, 119234, Russia; MD, PhD, Professor, Academician of the Russian Academy of Sciences, Head of the Hospital Surgery Department; I.M. Sechenov First Moscow State Medical University (Sechenov University), 8/2 Trubetskaya St., Moscow, 119991, Russia; PhD, Associate Professor, Human Anatomy and Histology Department; I.M. Sechenov First Moscow State Medical University (Sechenov University), 8/2 Trubetskaya St., Moscow, 119991, Russia; Associate Professor, Normal and Topographic Anatomy Department, Fundamental Medicine Faculty; Lomonosov Moscow State University, 1 Leninskiye Gory, Moscow, 119234, Russia; PhD, Head of the Department of Modern Biomaterials, Institute for Regenerative Medicine; I.M. Sechenov First Moscow State Medical University (Sechenov University), 8/2 Trubetskaya St., Moscow, 119991, Russia; PhD, Associate Professor, Human Anatomy and Histology Department; I.M. Sechenov First Moscow State Medical University (Sechenov University), 8/2 Trubetskaya St., Moscow, 119991, Russia; Associate Professor, Normal and Topographic Anatomy Department, Fundamental Medicine Faculty; Lomonosov Moscow State University, 1 Leninskiye Gory, Moscow, 119234, Russia; PhD, Associate Professor, Human Anatomy and Histology Department; I.M. Sechenov First Moscow State Medical University (Sechenov University), 8/2 Trubetskaya St., Moscow, 119991, Russia; Resident of the Student Scientific Circle, Human Anatomy and Histology Department; I.M. Sechenov First Moscow State Medical University (Sechenov University), 8/2 Trubetskaya St., Moscow, 119991, Russia; MD, PhD, Professor, General Director; Moscow Center for Health Problems under the Moscow Government, 14/3 Zhitnaya St., Moscow, 119049, Russia; PhD, Scientific Director of Biomedical Science & Technology Park; I.M. Sechenov First Moscow State Medical University (Sechenov University), 8/2 Trubetskaya St., Moscow, 119991, Russia; PhD, Associate Professor, Human Anatomy and Histology Department; I.M. Sechenov First Moscow State Medical University (Sechenov University), 8/2 Trubetskaya St., Moscow, 119991, Russia; Associate Professor, Normal and Topographic Anatomy Department, Fundamental Medicine Faculty; Lomonosov Moscow State University, 1 Leninskiye Gory, Moscow, 119234, Russia; Research Intern, Human Anatomy and Histology Department; I.M. Sechenov First Moscow State Medical University (Sechenov University), 8/2 Trubetskaya St., Moscow, 119991, Russia

**Keywords:** uncontrolled arterial hypertension, aortic arch aneurysm, direct mechanical testing methods, strength and elastic-deformative aorta characteristics, vascular wall stiffness, cardiovascular accident predictors

## Abstract

**The aim of the study** was to investigate the potential of direct mechanical testing methods in clinical practice to assess the strength and elastic-deformative characteristics of intraoperative samples of aortic arch aneurysm caused by uncontrolled arterial hypertension.

**Materials and Methods:**

The study experimental material was the resected parts of the aortic aneurysm obtained during aneurysm replacement surgery in a patient with uncontrolled arterial hypertension. The direct mechanical testing methods such as instrumental indentation and uniaxial extension were used.

**Results:**

It was shown that by the direct instrumental indentation it is possible to accurately assess and compare the stiffness of all three layers of the aortic wall. In this clinical case, the inner aorta layer was subject to the greatest atherosclerotic damage. In the media area, the values of this indicator were widely scattered, whereas the material was greatly dissected. By uniaxial extension method it is possible to obtain accurate parameters of the vascular wall strength, as well as to assess the stiffness, elasticity, and deformability of the intraoperatively resected aortic tissue. It was found that the aneurysm aortic wall, compared with the non-dilated aortic section, was characterized by a significantly lower strength in both longitudinal (by 4.25 times) and transverse (by 3.75 times) directions. In addition, aneurysm tissues demonstrated a significantly lower elasticity and deformability.

**Conclusion:**

The study demonstrated the perspectives and options of using in clinical practice current methods of direct mechanical testing, which makes it possible to obtain more accurate indicators of the strength and elastic-deformative vascular characteristics, to clarify the pathophysiological mechanisms of cardiovascular accidents, and to justify the need for regular monitoring of vascular wall stiffness in clinical practice, in particular in patients with uncontrolled arterial hypertension.

## Introduction

Currently, arterial hypertension is one of the most common cardiovascular diseases. According to a number of epidemiological studies, the incidence of high blood pressure (BP) in the adult population reaches 40%, whereas in the older age categories this value exceeds 80% [1–3]. It was shown that only 48% of patients are aware that they have this pathology. Approximately one third of those examined take antihypertensive drugs, and only 11% of them are really treated effectively. When the target BP is not achieved, the condition is called “uncontrolled arterial hypertension” [4].

High BP significantly increases the vascular accident risk and raises mortality rate from cardiovascular diseases by 8 times [5]. One of the serious complications of uncontrolled arterial hypertension is aortic aneurysm, which is often asymptomatic for a long time and is not detected by standard therapeutic examination [6–8]. The first signs of the existing pathology may be symptoms of a sudden acute aortic syndrome with the aorta dissection and rupture, the mortality of which is up to 90%. The life prognosis of such patients directly depends on the timely diagnosis and prevention of these emergency conditions [9–12].

To date, the main criterion for scheduled surgical treatment of aortic aneurysms is the aorta size combined with assessing the rate of increase in its diameter [13]. At the same time, much theoretical scientific evidence has been accumulated confirming the pathogenetic significance of vascular wall stiffness as an important independent predictor of vascular accidents [14–17]. In this regard, the non-invasive methods of indirect assessment of aortic wall stiffness, which primarily include ultrasound, magnetic resonance imaging and pulse wave velocity measurement, have been widely used in clinical practice in recent years [18–20].

More accurate indicators of physical and mechanical properties of biological tissues are provided by such direct mechanical testing methods as instrumental indentation and uniaxial extension, which are currently mainly used in studying animal model vessels, artificially grown experimental human tissues, and cadaveric material [21, 22]. These methods may be of particular significance in clinical practice. Thus, in uncontrolled arterial hypertension, of primary importance is measuring the physical and mechanical parameters of the aorta initial sections, in which the degree of atherosclerotic changes is maximum due to the greatest haemodynamic shock [23].

Thus, **the aim of this study** was to investigate the potential of direct mechanical testing methods in clinical practice to assess the strength and elastic-deformative characteristics of intraoperative samples of aortic arch aneurysm caused by uncontrolled arterial hypertension.

## Materials and Methods

The study experimental material included resected parts of the aortic aneurysm obtained as a result of open surgery in a patient with uncontrolled arterial hypertension.

*Patient S., female, aged 74, was admitted to the hospital surgery clinic of the N.V. Sklifosovsky Clinical Medicine Institute of the Sechenov University with complaints of weakness, dizziness, and more frequent events of increased blood pressure (up to 180/110 mm Hg). In anamnesis she suffered from hypertension for more than 30 years with a maximum increase in blood pressure up to 200/120 mm Hg, periodically took various antihypertensive drugs without effect. Three years before the admission, according to echocardiography, aortic dilation was frst identified, no surgical intervention was suggested. In July 2023, echocardiography revealed an increase in the diameter of the dilated aorta part to 68 mm. Consultation with a cardiac surgeon was recommended. In September 2023, the patient was consulted at the Sechenov University: echocardiography showed a dissecting aneurysm of the distal ascending aorta with the presence of parietal thrombomasses. The aorta diameter at the Valsalva sinus level was 33 mm, at the sino-tubular ridge level — 30 mm, the diameter of the distal ascending aorta part and the aortic arch reached 71 mm at a distance of 5.9 cm from the fibrous ring of the aortic valve (FRAV). Atherosclerotic lesions of the aorta root and walls, fibrous rings, aortic and mitral valve cusps, grade I aortic insufficiency, grade I mitral insufficiency, grade I tricuspid insufficiency, and type 1 diastolic left ventricle dysfunction were observed. The systolic function of the hypertrophied left ventricle was preserved (ejection fraction: 61%). There were no hypokinesia zones as well as evidence of pericardial or pleural effusion.*

*On admission: the patient condition was severe, hemodynamically stable. Grade II obesity. The heart area was unchanged, the heart rate — 84 per minute, blood pressure — 150/90 mm Hg. Pulse of satisfactory filling, arrhythmic. Heart tones were muffed, arrhythmic, no murmurs. Other organs and systems — without features.*

**Figure 1.**
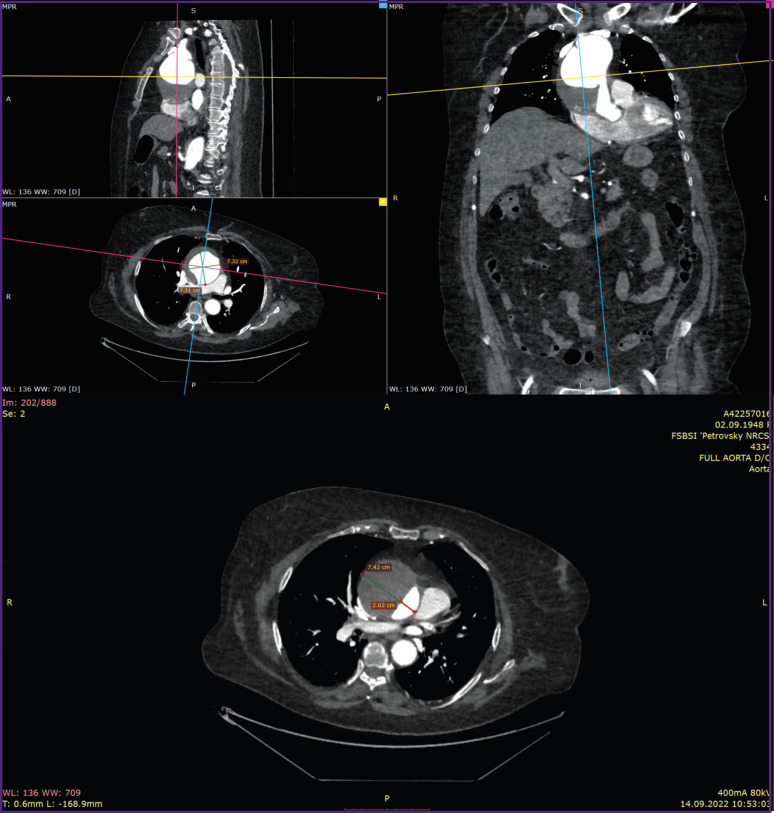
MSCT aortography

*MSCT aortography revealed an aneurysm of the distal ascending aorta and aortic arch up to 74 mm in diameter, and DeBakey type II aortic dissection (Figure 1). Doppler ultrasonography showed signs of diffuse atherosclerotic changes in the brachiocephalic arteries and aorta, as well as deformation of the aortic arch branches. Chest X-ray did not show focal or infiltrative shadows in the lungs. The pulmonary pattern was not enhanced. The lung roots were structured and not dilated. The diaphragm was of normal location. Sinuses were free. The aorta was dilated in the thoracic region, elongated, thickened with calcinosis. The heart was slightly enlarged in volume due to the left ventricle. Mediastinal shadow was not displaced. Electrocardiogram: sinus rhythm, heart rate — 96 per minute. The PQ interval — 0.20”, QRS — 0.09”, QRST — 0.34. The electrical heart axis was deviated to the left. Left ventricular hypertrophy and impaired left ventricular repolarization processes were revealed. Daily blood pressure monitoring was performed in outpatient conditions, on antihypertensive therapy. Stable moderate systolo-diastolic arterial hypertension with episodes of the BP increase up to a high level during physical exertion, with a violation of the circadian profile of diastolic BP of the non-dipper type, was revealed.*

*Complete blood count: HGB — 133 g/L, RBC — 4.71×10^12^/L, WBC — 8.8×10^9^/L, NEUT — 73.3%, LYMP — 17.5%, Mono — 6.7%, PLT — 275×10^9^/L. Biochemical blood test: total bilirubin — 9.2 μmol/L, direct bilirubin — 1.8 μmol/L, total protein — 75 g/L, urea — 8.9 mmol/L, creatinine — 92 μmol/L, GFR — 49.45 ml/min, glucose — 5.34 mmol/L, cholesterol — 6.72 mmol/L, triglycerides — 5.61 mmol/L, HDL-cholesterrol — 0.93 mmol/L, LDL-cholesterrol — 3.33 mmol/L, non-HDL-cholesterrol — 5.79 mmol/L, AST — 15 U/L, ALT — 13 U/L, CPK — 36 U/L, C-reactive protein — 8.1 mg/L, potassium — 4.6 mmol/L, sodium — 135 mmol/L, chloride — 101 mmol/L. Coagulogram: aPTT — 25.4 s, aPTT-PS — 0.82, PTT — 10.6 s, prothrombin (Quick activity) — 104.0%, INR — 0.97, fibrinogen — 5.39 g/L, antithrombin III — 131.0%, platelet aggregation — 76.4%. Common urine analysis revealed no pathologies.*

*Based on the examination data, the following diagnosis was established: “DeBakey type II aortic dissection, aneurysm of the distal ascending aorta and aortic arch. Atherosclerosis with the predominant damage to the aorta and brachiocephalic arteries. Hypertension of stage III, degree III, risk 4. Uncontrolled arterial hypertension. Hyperlipidemia. Left ventricular myocardial hypertrophy. Chronic kidney disease C3A stage. Hypercoagulation syndrome. Discirculatory encephalopathy stage II. Costitutional-exogenous obesity stage II”.*

*Preoperative transesophageal echocardiography: FRAV diameter — 31 mm. The diameter of the ascending aorta at the Valsalva sinus level — 30 mm, at the sino-tubular ringe level — 27 mm, 3.5 cm lower the FRAV — 40 mm, at the level of the distal section and arch — 74 mm. Aortic dissection at 4.5 cm from the FRAV with false channel thrombosis, atherosclerotic changes in the aortic root and walls, left ventricular myocardial hypertrophy. Local and global systolic function of the left ventricle was not impaired. Hemodynamic parameters and aortic valve function were normal. There was grade I tricuspid insufficiency. The descending aorta — without negative dynamics. For emergency life-saving indications, the patient underwent supracoronary prosthetics of the distal ascending part and semi-arch of the aorta with a synthetic 30 mm AlboGraft prosthesis using the Hemiarch technique under conditions of the antegrade monohemispheric brain perfusion, moderate hypothermia (27°C), artifcial blood circulation, and cardioplegia according to del Nido [24].*

**Figure 2.**
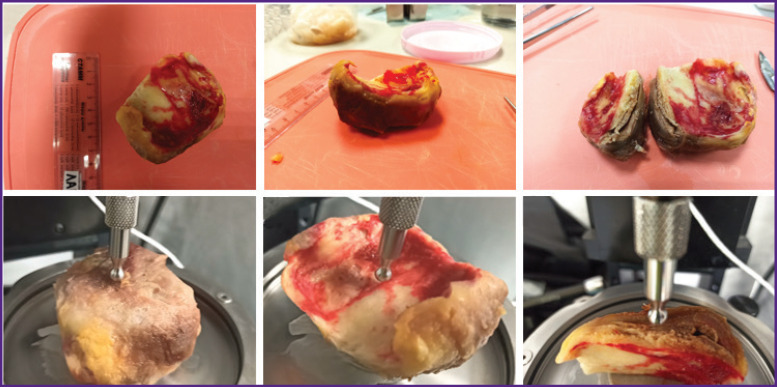
Instrumental indentation of aortic samples

The resected parts of the aortic aneurysm were delivered to the laboratory within 2 h after the replacement surgery. Instrumental indentation and uniaxial extension were used in the *in situ* experiment. Macroindentation was performed on a Mach-1™ v500csst universal micromechanical system (Biomomomentum Inc., Laval, Quebec, Canada). A metal spherical indenter with a radius of 3.25 mm was used. The samples were placed on a metal holder with their convex or concave side up, as well as with the cut side up to measure the inner layer. During all measurements, the material wetness was maintained by adding phosphate-buffered saline. Using the Find Contact function of the device, the sample was indented to a specified load (0.1 N), providing a pressing depth of approximately 0.5–1.0 mm (Figure 2).

To calculate the Young’s modulus, the obtained dependences of the load (*F*) on the indentation depth (δ) were approximated by the Hertz model:

F=43f(δ)E1−ν2δ32R,

where *E* — the Young’s modulus, **v** — the Poisson’s ratio of the sample, which for biological samples is considered equal to 0.5 [25]. A series of measurements (9-12) were performed on each side of the sample in the mapping mode; and the averaged data were analyzed.

**Figure 3.**
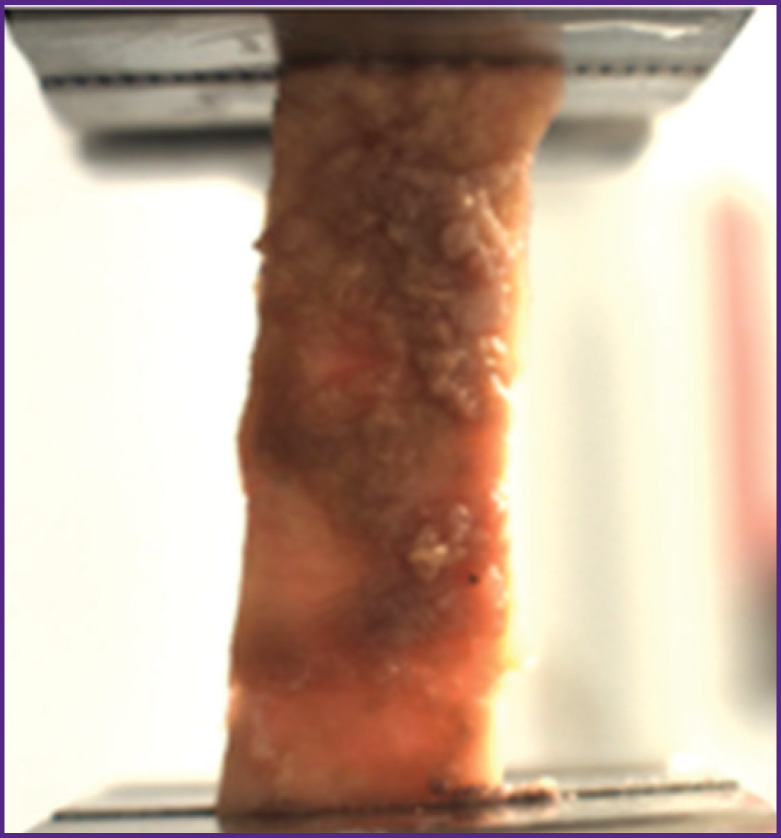
Uniaxial extension of aortic samples

For uniaxial extension testing, samples of 28.0×9.0 mm were cut in the directions along (n=9) and across (n=9) the blood fow using the standard cutting matrix. Samples of the aorta aneurysm and the aorta undilated section were examined by uniaxial tension until the rupture on the Mach-1™ v500csst universal micromechanical system (Figure 3). The material strength was assessed by the rupture stress index (σ, MPa):

σ=F/hw,

where *F* — the force during rupture, N; *h* — the average sample thickness, mm; *w* — the sample width, mm.

The deformability (ε, %) was determined by the value of the maximum elongation up to the rupture moment:

ε=ΔL/L,

where *ΔL* — the maximum sample elongation during the experiment, mm; *L* — the initial sample length, mm.

The material stiffness was defined by the values of the Young’s modulus (*E*, MPa):

E=σ/ε,

where *E* — the Young’s modulus, MPa; σ — the rupture stress index, MPa; ε — the maximum sample elongation before rupture moment, mm [22].

The study was approved by the Local Ethics Committee of Sechenov University in compliance with the ethical standards of the Helsinki Declaration (2013).

### Statistical data processing

The results of the study were processed by the method of variation statistics using the Microsoft Excel 2010 software. The normality of data distribution was checked by the Shapiro-Wilk criterion. Given that the distribution of the obtained values was normal, the mean values of indicators and their standard deviations were calculated. The statistical significance of differences in quantitative variables was assessed by Student’s t-criterion. The results were considered statistically significant at p<0.05.

## Results

**Figure 4.**
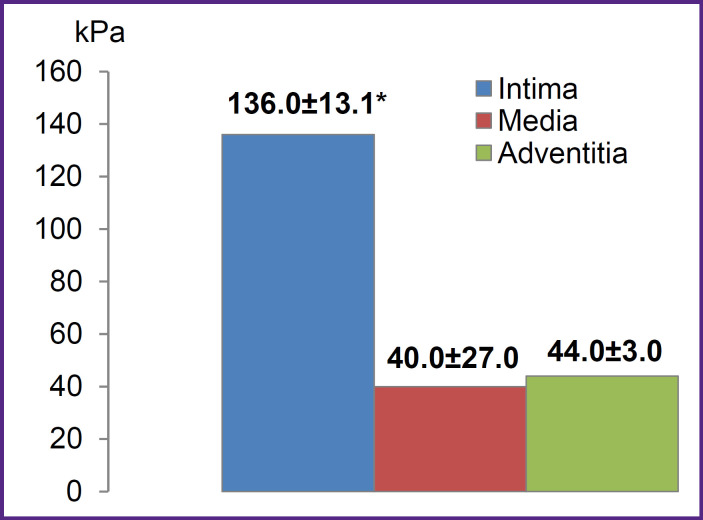
Young’s modulus of the aortic aneurysm wall * p<0.05

The wall of the aorta, as an elastic type vessel, has a three-layer structure consisting of the inner layer (intima), media and adventitia [26]. It was shown that the inner layer of the resected aorta section (concave side) had significantly higher stiffness (Young’s modulus) compared to the outer (convex) side and the medium layer (Figure 4).

It should be noted that studying the media in the area of the maximum aortic dilation showed a large spread of values of this indicator and stratification of the investigated material.

**Figure 5.**
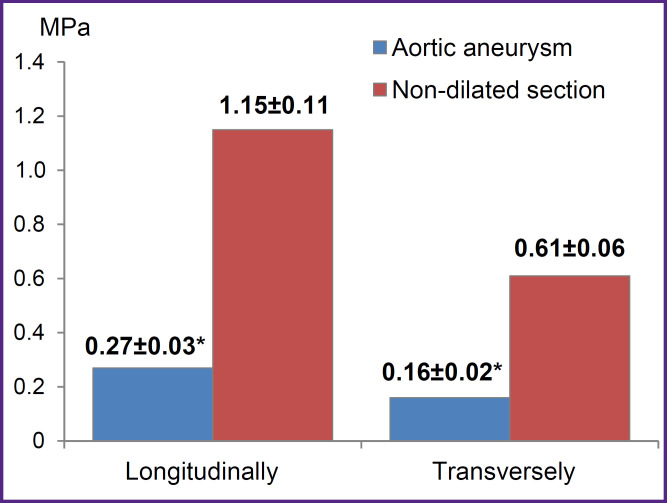
Maximum aortic samples extension at rupture * p<0.05

According to the uniaxial extension data, the wall of the aneurysmal aorta sac, compared to the non-dilated aortic section, was characterized by significantly lower strength in both longitudinal (by 4.25 times) and transverse (by 3.75 times) directions, which was indicated by significantly lower values of the maximum rupture stress (σ, MPa) (Figure 5).

**Figure 6.**
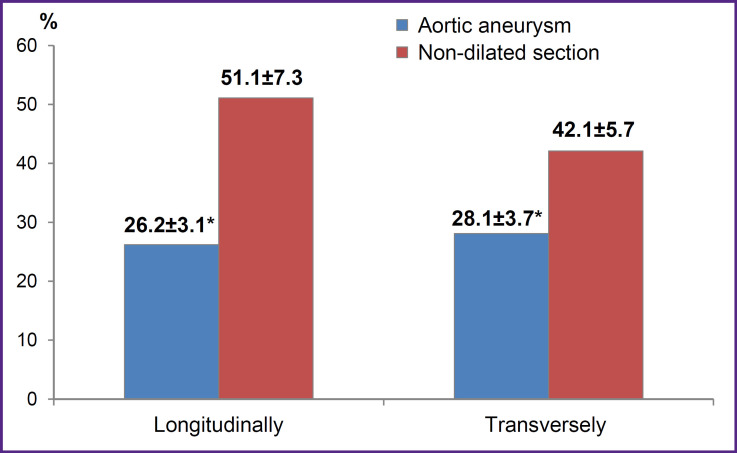
Maximum aortic samples extension before rupture * p<0.05

Aneurysm tissues demonstrated significantly lower elasticity and deformability in terms of maximum sample elongation before the rupture (ε, %) (Figure 6).

**Figure 7.**
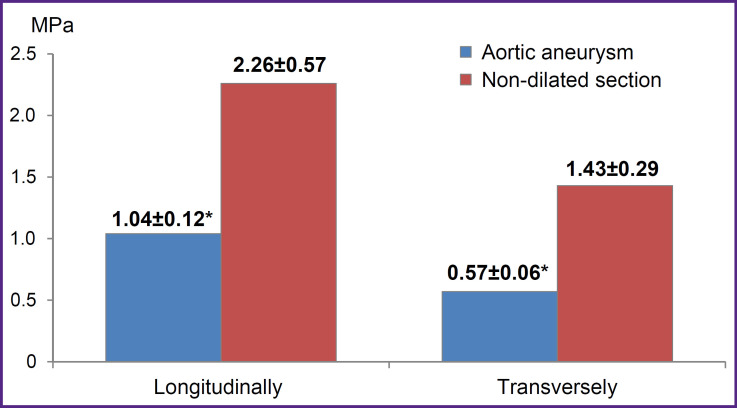
Young’s modulus (stiffness) of aortic samples * p<0.05

The Young’s modulus in the dilatation aorta area had lower values compared to the non-dilated aorta section (*E*, MPa) (Figure 7).

## Discussion

Aortic aneurysm complicated by lightning-fast dissection and rupture of the vascular wall is a serious medical and socio-economic problem in most countries of the world. In uncontrolled arterial hypertension, changes in strength and elastic-deformative characteristics are noted mainly in the aortic arch, which is subject to maximum blood pressure fuctuations and makes the greatest contribution to the transformation of pulsating blood fow due to equal extension and compression to original size [5, 23, 26–28]. Under conditions of increased hemodynamic shock, there is an increase in intima permeability for low-density lipoproteins and their deposition in the aortic wall, which is accompanied by a significant change in the strength and elastic-deformative characteristics of this vessel [5–8]. Hence, the methods of indirect blood vessel stiffness assessment demonstrated that vascular wall stiffness is an important independent vascular accident predictor [14–17].

This clinical case demonstrated the perspectives and options of current methods of direct mechanical testing in clinical practice in assessing physical and mechanical properties of intraoperatively taken aortic material. Thus, it was shown that by the direct instrumental indentation it is possible to accurately assess and compare the stiffness of all three layers of the aortic wall (the median layer was assessed for the first time), which in the future, with a sufficient sample, will allow to clarify pathophysiological mechanisms of aneurysm formation. In this case, it was established that the inner vessel layer was subject to the greatest atherosclerotic damage, inflammation in which was accompanied by release of many biologically active substances and disorganization of the connective tissue structures of the aorta media, which is the main risk factor for development of aortic aneurysm and its dissection. Hence, a wide variation of Young’s modulus values and stratification of the investigated material was observed during studying media in the aortic dilation section.

Accelerated formation of diffuse-focal intima thickening in this aorta section caused by uncontrolled arterial hypertension serves as a morphological basis for interrelated destructive and compensatory-reparative processes leading to the loss of aortic wall elasticity, aneurysm formation, and aortic layer thinning [5–8, 15, 17, 27]. Thus, it was shown that by uniaxial extension method it is possible to obtain accurate parameters of the vascular wall strength, as well as to assess the stiffness, elasticity, and deformability of intraoperatively taken aortic tissue. In this case, it was found that the aneurysm aortic wall, in comparison with the non-dilated aortic section, was characterized by significantly lower strength, lower elasticity and deformability. Obtaining true values of aortic wall stiffness by the uniaxial extension method with a sufficient sample and their comparison with the results of preoperative studies of these vessels by indirect assessment methods will make it possible to evaluate the reliability level of the latter methods, to create a mathematical model of the correspondence between the direct and indirect measurement data for predicting the risk of aneurysm dissection and rupture, as well as for conducting a timely scheduled surgery.

In addition, revealing the predominantly damaged layer in the aortic wall can determine the differentiated approach to development of an optimal constructive and reparative management of aneurysm prosthetics, which will contribute to vascular sutures fixation and anastomosis integrity in the postoperative period. Only clear interaction between cardiologist, functional diagnostic physician, and a cardiovascular surgeon will make it possible to avoid errors in assessment of the disease prognosis and to choose the only correct tactics for managing such patients.

## Conclusion

The study demonstrated the perspectives and options of such current methods of direct mechanical testing as instrumental indentation and uniaxial extension in clinical practice. Using these biomedical technologies in surgery-related clinical studies makes it possible to obtain more accurate indicators of the strength and elastic-deformative vascular characteristics, to clarify the pathophysiological mechanisms of cardiovascular accidents, and to justify the need for regular monitoring of vascular wall stiffness in clinical practice, in particular in patients with uncontrolled arterial hypertension. This will contribute to development of optimal management tactics for such patients, prevention of life-threatening conditions and mortality reduction.
